# Strengthening genomic surveillance for neglected tropical diseases in the Americas

**DOI:** 10.1016/j.lana.2025.101338

**Published:** 2025-12-12

**Authors:** The Lancet Regional Health – Americas

The American continent faces a silent inequity: while some infectious diseases (such as arboviruses) receive significant attention and funding resources, others remain overlooked, despite their profound impact on society. Among these, Neglected Tropical Diseases (NTDs; a diverse group of infections defined by WHO that cause major health, social, and economic burdens in tropical and subtropical regions) stand out for their complex and dynamic profiles. WHO estimates that NTDs cause around 120,000 deaths and 14.1 million lost disability-adjusted life years each year, and more than 1.49 billion people globally need annual care for at least one NTD. This burden can cost developing countries billions of US$ in health expenses and lost productivity. According to the Pan American Health Organization (PAHO), 50 million people in the Americas are affected by NTDs.

NTDs involve a variety of hosts, transmission cycles, and diverse subpopulations of etiological agents, all of which complicate diagnosis and treatment. Persistent gaps—such as underdiagnosis, delayed outbreak detection, and limited understanding of transmission dynamics—challenge effective control and elimination efforts. Genomic surveillance has emerged as a tool to transform our understanding of these complex and often neglected diseases, enabling more precise detection and tracking of variants and drug resistance, and ultimately supporting more effective and equitable public health interventions.

Genomic surveillance comprises the continuous analysis of pathogens’ genetic material and enables researchers to identify variants, track transmission, monitor drug resistance, and anticipate outbreaks. In this December issue of *The Lancet Regional Health—Americas*, Walter and colleagues (2025) used genomic sequencing to analyze tuberculosis transmission dynamics in Brazilian prisons; in another study, also in Brazil, Parreiras de Jesus and colleagues identified the simultaneous circulation of dengue and chikungunya viruses, informing targeted public health responses. Although are not examples of NTDs, a genomic study of *Leishmania infantum* uncovered markers of drug resistance, an important element of pathogen surveillance. This highlights the value of genomic surveillance and the need to expand its use for a wider range of NTDs in the Americas.

The USA stands out as an example of well-conducted genomic surveillance, where continuous investments and integration between research centers—both domestic and international—and public agencies have been key. The Centers for Disease Control and Prevention (CDC) stands as a model organization, providing robust monitoring that enables rapid response to emergencies and identification of new threats, being a reference to the Americas. However, recent developments at the CDC, such as budget constraints and organizational restructuring, have led to uncertainty regarding the agency's future role in national and regional health security, undermining disease surveillance, global health expertise, and outbreak response. The CDC has long played a crucial role in regional collaborations, sample processing, and training, supporting public health outside the USA. Its reduced capacity and removal of key data will leave the region more vulnerable to emerging health threats and weaken global health security.

Latin America and the Caribbean, where NTDs are common, face substantial inequalities, including genomic surveillance. The CABANA consortium from Costa Rica, VigiGenômica from Brazil and the PAHO Genomics Surveillance Regional Network are examples of notable progress, even in the face of difficult circumstances. However, the long-term sustainability of these initiatives depends on continuous funding, institutional recognition and support, and policies that recognize and value science. Brazil, the largest country and a reference for the region, has experienced considerable federal budget limitations in the areas of science and public health. Economic uncertainty and low political prioritization make it difficult to maintain a long-term project and undermine progress in areas that require continuous and stable investment. Although collaboration is common and essential in research, it is also crucial for the region to strengthen regional leadership and autonomy through diversified and domestic partnerships.

Although there are a great number of research centers and some genomic surveillance initiatives in Latin America and the Caribbean, access to them is still limited, especially outside major urban centers and in remote areas. Also, most initiatives are focused on arboviruses and SARS-CoV-2. Genomic surveillance for schistosomiasis, soil-transmitted helminthiases (such as ascariasis and trichuriasis), lymphatic filariasis, onchocerciasis, and other parasitic and vector-borne diseases (such as bartonellosis and myiasis) are still scarce. Most available data come from isolated academic studies rather than coordinated, ongoing surveillance programs. Strategies such as platforms and data sharing could be adopted to expand the reach of individual surveillance efforts.

Nevertheless, the prioritization of NTDs for genomic surveillance is shaped by both epidemiological and economic factors. Infection diseases with greater visibility, such as malaria and arboviruses, usually receive more resources and attention, while NTDs—which affect mostly rural and other systematically marginalized populations—receive lower or no attention. This unequal prioritization perpetuates the cycle of invisibility of NTDs and limits scientific, technological, and clinical progress. A comment published by Salvato and colleagues discussed this gap for tuberculosis—although it is not considered an NTD—and suggests the pathway for genomic surveillance implementation. This could be extended to NTDs as they are subject to similar barriers.

Decentralizing genomic surveillance is an urgent priority. Expanding access to sequencing and analysis tools is essential for making innovation more accessible to all. Initiatives such as itinerant laboratories and portable technologies enable real-time sample sequencing, while training local teams and engaging local governments and communities could ensure more reliable results and efficient workflows. These could not only accelerate diagnosis and decision-making but also enhance local expertise and address the unique needs of each territory.

Strengthening genomic surveillance for NTDs in the Americas is both urgent and strategic. Investing in infrastructure, decentralizing tools, and empowering professionals are essential steps to overcome historical inequalities and provide effective response for the most vulnerable populations. Regional collaboration, support from financial agencies, and active engagement of the scientific community are fundamental to transforming the promise of genomic surveillance into real public health impact. In line with the WHO's 2021–2030 road map for NTDs, genomic surveillance can be crucial to ending neglect and advancing health equity. While some steps have already been taken, much more remains to be done.

